# Beta Irradiation Effects on Electrical Characteristics of Graphene-Doped PVA/n-type Si Nanostructures

**DOI:** 10.1021/acsomega.3c08449

**Published:** 2024-05-20

**Authors:** Özlem Abay, Murat Ulusoy, Esra Uyar, Uğur Gökmen, Sema Bilge Ocak

**Affiliations:** † Graduate School of Natural and Applied Sciences, Department of Advanced Technologies, Gazi University, 06500 Ankara, Turkey; ‡ Turkish Energy, Nuclear and Mineral Research Agency, 06980 Ankara, Turkey; § Faculty of Science, Department of Physics, Gazi University, 06500 Ankara, Turkey; ∥ Faculty of Technology, Department of Metallurgical and Materials Engineering, Gazi University, 06500 Ankara, Turkey

## Abstract

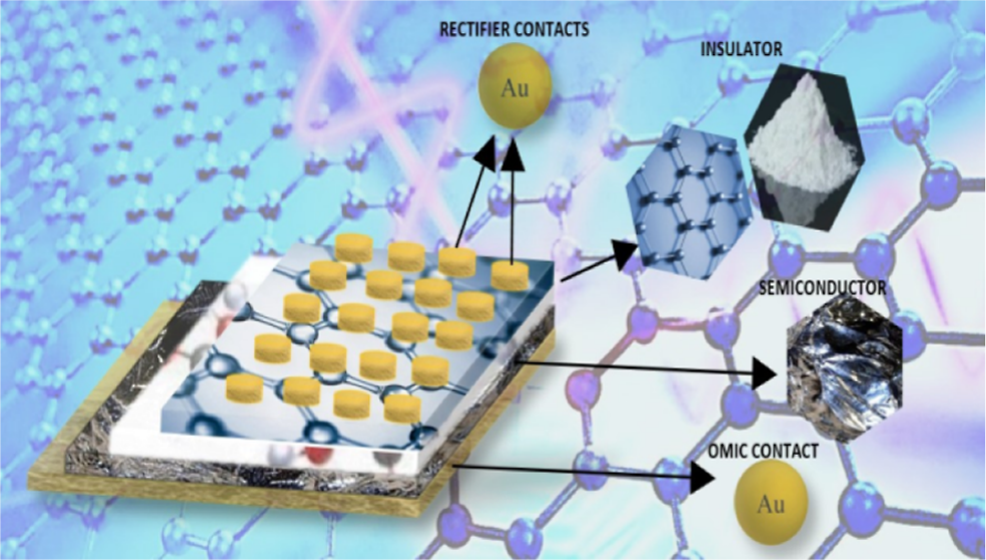

This study investigates the beta irradiation’s impact on the electrical features of interfacial nanostructures composed of poly(vinyl alcohol) (PVA) doped with graphene. The integration of graphene, a 2D carbon allotrope renowned for its exceptional electrical conductivity, into PVA nanostructures holds significant promise for advanced electronic applications. Beta irradiation, as a controlled method of introducing radiation, offers a unique avenue to modulate the properties of these nanostructures. Therefore, this study examines the Au/3% graphene(Gr)-doped PVA/n-type Si structure with and without beta (β) radiation. The effect of beta radiation on the electrical properties of the Au/3% graphene(Gr)-doped PVA/n-type Si structure has been researched by utilizing the current–voltage (*I–V*) data. The studied structures were exposed to a ^90^Sr β-ray source at room temperature to show the effect of beta radiation. The series resistance (*R*_s_), shunt resistance (*R*_sh_), ideality factor (*n*), barrier height (BH) (Φ_B0_), and saturation current (*I*_o_) were computed using the *I*–*V* data after ^90^Sr β-ray irradiation (0, 6, and 18 kGy) and before using the thermionic emission, Norde, and Cheung methods. The BH, ideality factor, and series resistance were calculated using the *I*–*V* data as follows: 0.888 eV, 3.21, and 5.25 kΩ for 0 kGy; 0.782 eV, 5.30, and 3.47 for 6 kGy; 0.782 eV, 5.46, and 2.63 kΩ for 18kGy. The BH, ideality factor, and series resistance were also calculated using the Cheng Methods, and the following results were found respectively: 7.22, 0.74, and 3.97 kΩ (Cheng I), and 3.22 kΩ (Cheng II) for 0 kGy; 5.14, 0.813, and 2.72 kΩ (Cheng I), and 2.14 kΩ (Cheng II) for 6 kGy; 6.78, 0.721, and 1.96 kΩ (Cheng I), 1.64 kΩ (Cheng II) for 18 kGy. The BH and series resistance were defined as 0.905 and 16.12 kΩ for 0 kGy, 0.859 and 5.31 kΩ for 6 kGy, and 0.792 and 2.49 kΩ for 18 kGy, respectively. Interface states density (*N*_ss_) as a function of *E*_c_–*E*_ss_ was also attained by taking into account the voltage dependence of *n*, Φ_B_, and *R*_s_. Experimental results showed that the values of *n* and *N*_ss_ increased with an increase in the β-ray radiation dose. On the other hand, the saturation current (*I*_o_), Φ_B0_, and *R*_s_ values decreased with the increase in the β-ray radiation dose. The obtained results indicate a nuanced interplay between β irradiation dose and the nanostructure’s overall electrical properties. Insights gained from this study contribute to the understanding of radiation-induced effects on graphene-doped polymer nanostructures, providing valuable information for optimizing their performance in electronic applications.

## 1 Introduction

The semiconductor industry has spent the last 40 years trying to form nanoelectronic devices with improved performance. Semiconductor radiation detectors, which are used for various applications and fabricated from costly high-purity inorganic crystals, such as germanium and silicon, ensure higher energy resolution compared to radiation detectors of other types. In recent years, organic semiconductors have facilitated the development of various organic electronic devices in the traditional electronics fields.^1−6^ There has been growing interest in exploring novel materials and nanostructures for advanced electronic applications, driven by the continuous quest for improved device performance and functionality. Graphene, a hydrogenated derivative of graphene, has emerged as a promising candidate due to its unique electronic properties and compatibility with various substrates. Allotropes of carbon-based nanomaterials with distinctive electrical characteristics, such as carbon nanotubes (CNT) and graphene foils, have been developed for commercial use.^7^ Application to technologies for radiation detection has also been encouraged.^8^ The electrical conductivity per unit charge, which is defined by charge carrier mobility, exhibits a considerable effect on the sensitivity of radiation detectors.^7−11^ One such intriguing application involves the integration of graphene into poly(vinyl alcohol) (PVA)/n-type silicon (Si) nanostructures, offering a synergistic platform for innovative electronic devices.

Organic synthesis allows for the versatile design of a broad spectrum of organic semiconductors, presenting several advantages due to their noncorrosive nature, flexibility, and cost-effectiveness. These attributes make organic semiconductors particularly appealing for applications for which traditional materials, such as metals, may be less suitable. In large-area electronic devices, such as capacitors and organic electroluminescence (EL) devices, the use of organic semiconductors can contribute to cost reduction and increased flexibility, facilitating their incorporation into diverse environments.^12−15^

Despite these advantages, the proliferation of electronic devices in various settings exposes them to external factors including radiation. Beta irradiation, characterized by the emission of high-energy electrons, poses a notable environmental challenge that electronic components may confront. The intricate interaction between beta radiation and nanostructured materials can lead to complex effects on the electrical characteristics of these materials. Such effects have the potential to influence the overall performance and reliability of electronic devices.

As electronic technologies continue to advance, understanding the impact of radiation on novel materials, especially organic semiconductors, becomes imperative. Investigating the response of organic semiconductors to beta irradiation is crucial for evaluating their suitability in radiation-prone environments and optimizing their performance under such conditions. This exploration not only contributes to the fundamental understanding of the behavior of organic semiconductors under radiation exposure but also guides the development of radiation-resistant electronic components for diverse applications. It is anticipated that organic semiconductors will contribute to the development of low-cost and flexible radiation detectors with large detection zones. Several studies have been carried out to develop a real-time radiation detector using the electroconductive polymer polyaniline (Pani). The real-time radiation detector was successful in detecting the α and β particles.^16,17^ However, the sensor sensitivity of the device is still weak. Especially, its sensitivity for detecting β particles must be developed before it can be used for practical purposes. Among the various polymeric materials, poly(vinyl) alcohol (PVA) is a water-soluble, nontoxic, and semi-crystalline polymer. Moreover, the PVA polymer has a wide range of crystallinity, low conductivity, good charge storage capacity, very high dielectric strength, and interesting physical characteristics. In this regard, PVA is one of the most significant polymer materials used as an organic interface material in the construction of electronic devices. High conductivity, low dielectric loss, and high dielectric constant can be achieved by alloying the PVA polymer with suitable metals or graphene.

Metal–polymer/insulator–semiconductor (MPS/MIS) devices have interfacial states (*N*_ss_), series resistance (*R*_s_), the series resistance, and the interfacial layer, and the voltage applied to this material is dealt with over the layer of depletion. The electrical characteristics of these structures are therefore different from their ideal behavior under conditions such as lighting or radiation. Namely, the optical and electrical properties of the MPS/MIS structures are significantly dependent on *N*_ss_, *R*_s_, and radiation as well as the applied electric field, the doping, and the interfacial layer of the semiconductor devices. However, the surface states between the interfacial layer and semiconductor act as recombination centers, and they are capable of capturing or releasing electrical charges under the radiation or electric field. Such recombination centers also offer a tunnel road for the carriers. Though electron–hole pairs are formed by radiation in the semiconductor, only the holes can diffuse into the interface layer because their mobility is smaller compared to electrons that get out of the interface or recombine with holes. On the contrary, the radiation does not have adequate energy to generate electron–hole pairs, so many energetic particles and photons go right through the device without being absorbed by the semiconductor devices.^18,19^ Until now, ionizing radiation’s effects on semiconductor devices have already been the subject of several studies.^20−22^ However, the literature has a limited number of studies on the consequences of simultaneous voltage and radiation alterations. Furthermore, the effect of radiation on materials varies significantly if different materials are used as interlayers or if the dose and/or type of radiation varies.

MS contacts are crucial and good experimental instruments for researching the interactions and effects of ionizing radiation on interfaces despite the radiation hardness testing and reliability concerns. Different radiation types, such as swift heavy ions (SHI), cosmic rays, and low energy ions (γ rays, protons, alpha particles, neutrons, and electrons), have different effects on electronics via various methods. High-energy ions interact with the target substance and lose most of their energy there. Elastic collisions (atomic energy loss) are observed toward the entry of the ion field first, and then inelastic collisions (electron energy loss) are observed close to the entrance. Each type of energy loss mechanism has a different impact on the interface. Nuclear energy loss results in sporadic faults at interfaces, whereas electronic energy loss forms zones of complex faults in the materials, and it is also known to result in the annealing of faults.^23−25^ The motivation behind investigating the beta irradiation on the electrical characteristics of graphene-doped PVA/n-type Si nanostructures for radiation sensors lies in the imperative to enhance the performance and reliability of radiation detection technologies. Beta irradiation, characterized by the emission of high-energy electrons, represents a significant environmental challenge that electronic components, particularly radiation sensors, may encounter. By studying the effects of beta irradiation on the electrical characteristics of graphene-doped PVA/n-type Si nanostructures, researchers aim to gain insights into how these materials respond to radiation exposure. This understanding is crucial for optimizing the design and composition of radiation sensors, ensuring their accuracy, sensitivity, and stability in real-world applications. The research contributes not only to advancements in nanotechnology but also to the development of radiation sensors that play a vital role in various fields including nuclear facilities, medical settings, and environmental monitoring, ultimately enhancing safety and reliability in radiation detection.

The motivation behind the study is to explore and understand how beta irradiation affects the electrical characteristics of a specific type of nanostructure: graphene-doped PVA/n-type Si nanostructures.

To our knowledge, the literature does not have a detailed study on the impact of ^90^Sr beta (β) rays on the electrical properties of the Au/n-Si structures with Gr-doped PVA interfacial layers. This study aims to obtain high-performance Au/n-type Si structures with a 3% Gr-doped PVA interfacial layer after and before ^90^Sr β-ray irradiation. To investigate the effect of the ^90^Sr β-ray irradiation on these structures’ electrical features, we measured the *I*–*V* properties before and after exposing them to the ^90^Sr β-ray irradiation at room temperature. We calculated the values of the series resistance, ideality factor, interface state, saturation current, shunt resistance, and barrier height (BH) using the standard, Cheung, and Norde functions for the cases before and after ^90^Sr beta (β) irradiation. Then, we compared these values.

## 2 Experimental Details

**Figure 1:**
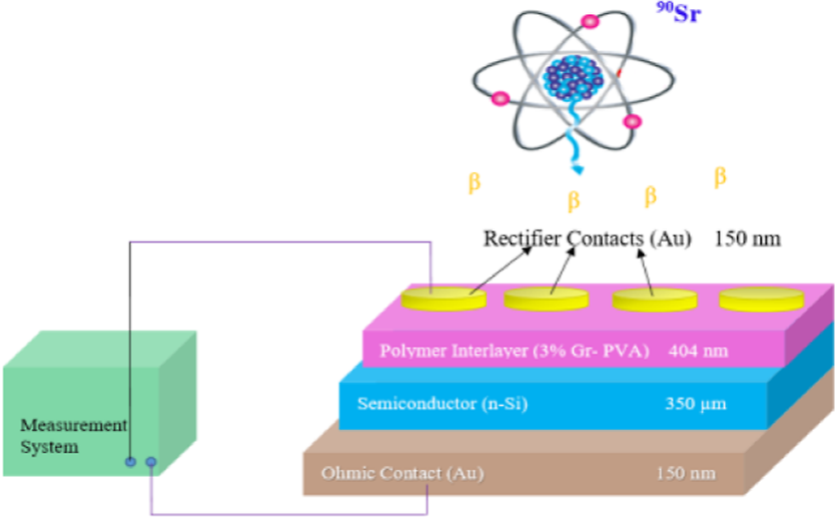
Au/3% Gr-doped PVA/n-type Si structures schematic diagram.

Au/3% Gr-doped PVA/n-type Si was grown on the phosphor-doped (4.3 × 10^15^ cm^–3^) n-type Si wafer with an orientation of (100), diameter of 2 in., thickness of 350 nm, doping donor atoms (*N*_C_) of 2.8 × 10^19^ cm^–3^, and resistivity of 0.5 Ω cm. Details about the chemical cleaning processes of the n-Si wafer, as well as the formation and deposition of the doped-polymer (Gr-PVA) organic thin film, were provided in the previous study.^26^Figure 1 presents the schematic diagram of the fabricated Au/3% Gr-doped PVA/n-type Si (MPS) structure. Keithley 2400 Source Meter was used to measure the *I*–*V* values for revealing the structure’s electrical properties at room temperature. Then, the Au/3% Gr-doped PVA/n- type Si (MPS) structure was exposed to high radiation with 6 and 18 kGy using ^90^Sr β-ray irradiation. Finally, the *I*–*V* values of these structures were measured again using the same Keithley 2400 Source Meter.

## 3 Results and Discussion

**Figure 2:**
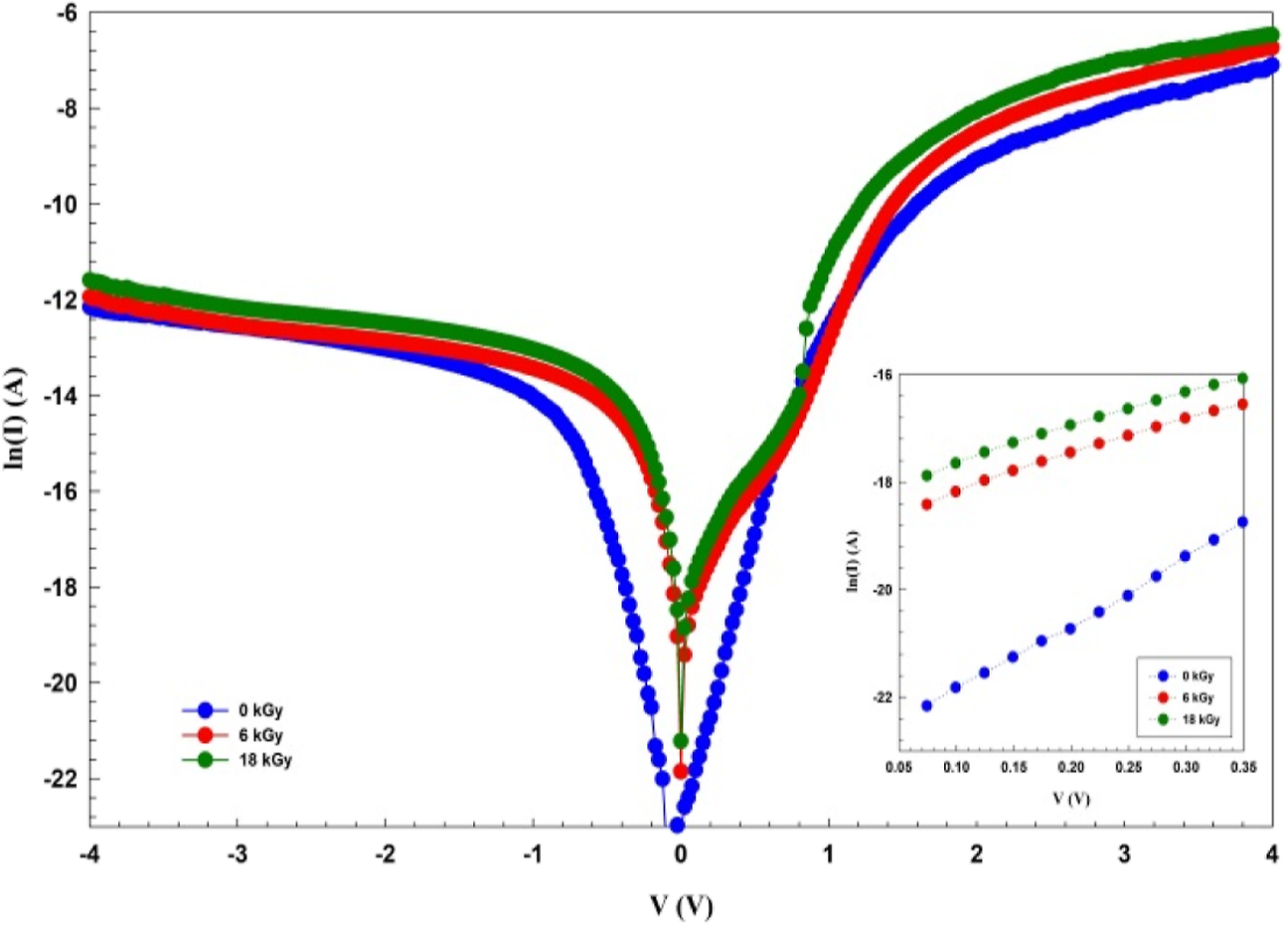
ln *I* versus *V* plots of Au/3% Gr-doped PVA/n-type Si diode.

The electrical properties of PVA doped with 3% graphene nanostructure are defined using the thermionic emission (TE) theory.^18^ The widely adopted approach for facilitating the passage of high-energy electrons over the BH (Φ_B0_) in metal–semiconductor (MS) type Schottky diodes (SDs), whether equipped with or without an interlayer at the M/S interface, is the standard TE theory. In accordance with this theory, when the SD (SD) experiences *V* ≥ 3*kT*/*q*, the relationship between forward-bias voltage and current can be expressed as follows
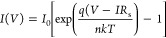
1
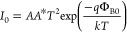
2
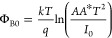
3
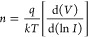
4where *q*, *A*, A*,*IR*_s_, *k*, *I*_0_, and Φ_B0_ denote the electron charge, rectifier contact area (7.85 × 10^–3^ cm^2^), the effective Richardson-constant (=112 A/cm^2^ K^2^ for n-type Si), the voltage-drop on the *R*_s_ (series resistance), Boltzmann constant, reverse saturation current, and the bias BH, respectively. First, the *I*_0_ values were computed for the points at which the ln *I*–*V* curves intersect the ln *I* axis. The linear ranges of *I*_0_ have been defined before and after beta (β) irradiation from approximately 0.05 to 0.35 V. The slopes of the linear parts of the ln *I*–*V* curves were calculated by utilizing the reciprocal of the thermal energy (*kT*/*q*), and the electrical parameter *n* was found to deviate significantly from the TE theory. The semilog *I*–*V* curves of Au/%3 Gr-doped PVA/n-Si structures before and after beta (β) irradiation (0, 6, 18 kGy) are illustrated in Figure 2 over a large voltage range (±4 V). As shown in the figure, all the ln *I*–*V* curves indicate good rectification behavior. While the curves behave linearly well at lower positive voltages, there are bends at higher positive voltages. These bends are caused by the *R*_s_ and the organic (Gr:PVA) interface layer.

It is generally assumed that radiation has two types of effects on materials: permanent effects and temporary effects.^18^ The temporary effect causes electron–hole pair recombination or radiation-induced generation. On the other hand, the permanent effect, which leads to a change in the crystal lattice structure, is mostly due to radiation bombardment. Besides, irradiated semiconductor devices might have eight effects, where one or a combination of them might have an effect.^27^ Eventually, radiation-induced defects, surface traps, recrystallization, dangling bonds, etc. greatly affect the performance of irradiated devices. The measured values must be analyzed in detail for a precise and comprehensive understanding of these effects. Therefore, the effect of radiation on n-Si type SDs doped with 3% Gr-doped PVA was investigated using the *I*–*V* characteristics. Figure 2 presents the Au/%3 Gr-doped PVA/n-Si structure’s *I*–*V* characteristics before irradiation and after different β-irradiation doses (0, 6, and 18 kGy). The linear components of forward bias in this figure also indicate the ln (*I*) – *V* properties, which are used in the calculations. The currents are graphed on a logarithmic plot to estimate forward and reverse currents together. The structures in Figure 2 display leakage and current normal rectification behavior with a low turn-on voltage.

**Figure 3:**
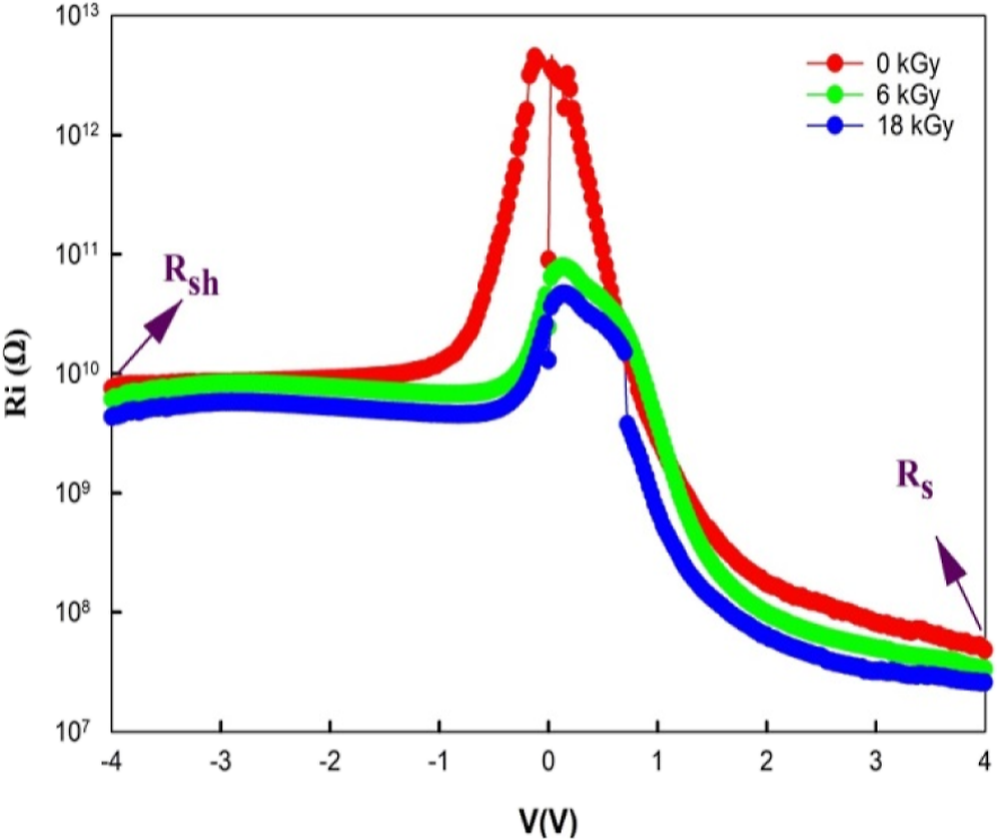
*R*_i_ versus *V* plots of the Au/3% Gr-doped PVA/n-type Si diode.

The simplest form of Ohm’s Law was used to derive *R*_s_ and *R*_sh_ values from the *I*–*V* data and plot in Figure 3. Table 1 shows the values of *n*, *I*_0_, Φ_B0_, *R*_sh_, *R*_s_, and Rectifier rate (RR = *I*_forward_/*I*_reverse_ at ±4 V) of the %3 Gr-doped PVA interfacial layer structures for the cases before and after (β) ray irradiation. The table indicates that *n* values are greater than unity due to *N*_ss_, BH image power reduction, voltage-dependent Φ_B0_, interfacial layer, and spatial barrier inhomogeneity in the M/S interface. The electron–hole pair generation under the impact of radiation leads to the increase in *I*_o_ with β radiation.

**Table 1:** Au/3% Gr-Doped PVA/n-type Si Structure’s Electrical Parameters Before and After Beta (β) Irradiation Doses

β-irradiation doses (kGy)	*n*	*I*_0_ (A)	Φ_B0_ (eV)	*R*_s_ (kΩ) (+4 V)	*R*_sh_ (MΩ) (−4 V)	RR
0	3.21	9.86 × 10^–11^	0.888	4.85	0.755	155.47
6	5.30	5.91 × 10^–9^	0.782	3.36	0.613	182.45
18	5.46	1.03 × 10^–8^	0.768	2.59	0.433	167.41

Since the field in the reverse bias zone is far stronger than that in the forward bias zone, it can prevent electron–hole (e–h) pairs’ recombination, which happens during radiation. Even though the *R*_sh_ value increases with the decrease in β-irradiation, *R*_s_ becomes almost constant. On the other hand, a reduction in layer width and a consequent rise in radiation may be the cause of the drop in BH with rising radiation.^28^ All the results in Table 1 suggest that β-irradiation is more useful for operating various types of electronic equipment.

**Figure 4:**
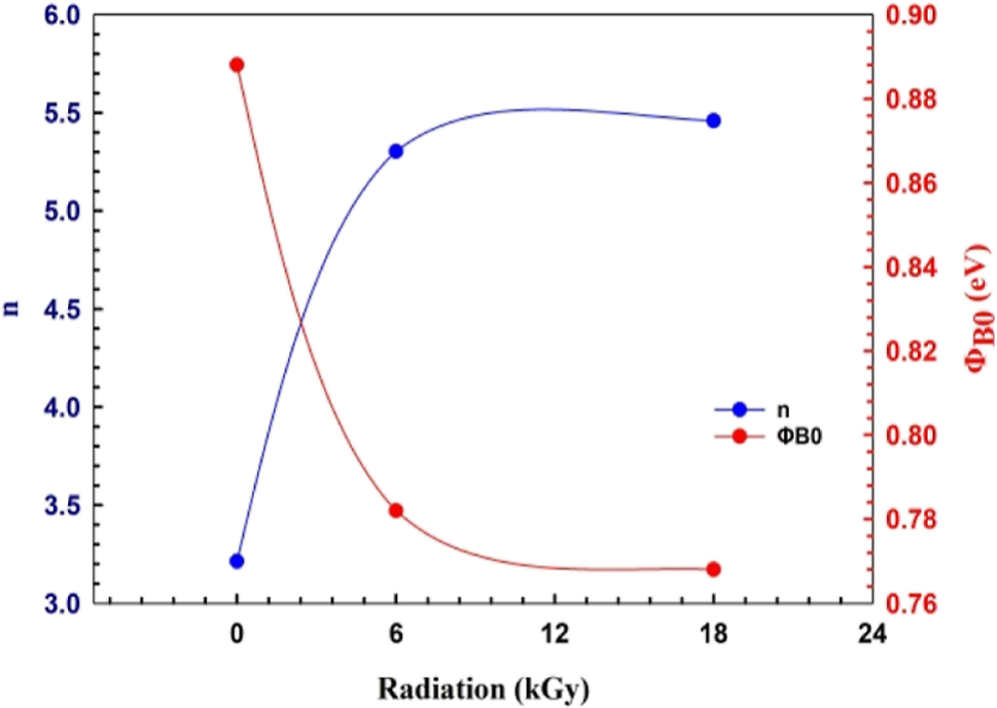
BH and ideality factor depend on β-irradiation.

The BH and ideality factor are depicted in Figure 4 as a function of β-irradiation. The *n* values that were determined before and following various dosages of β-irradiation are substantially greater than one. This is because, in addition to TE theory, there are other potential carrier processes (surface state-induced tunneling, diffusion theory, BH, Gaussian distribution, dislocations, and generation-recombination theory).^29^

**Figure 5:**
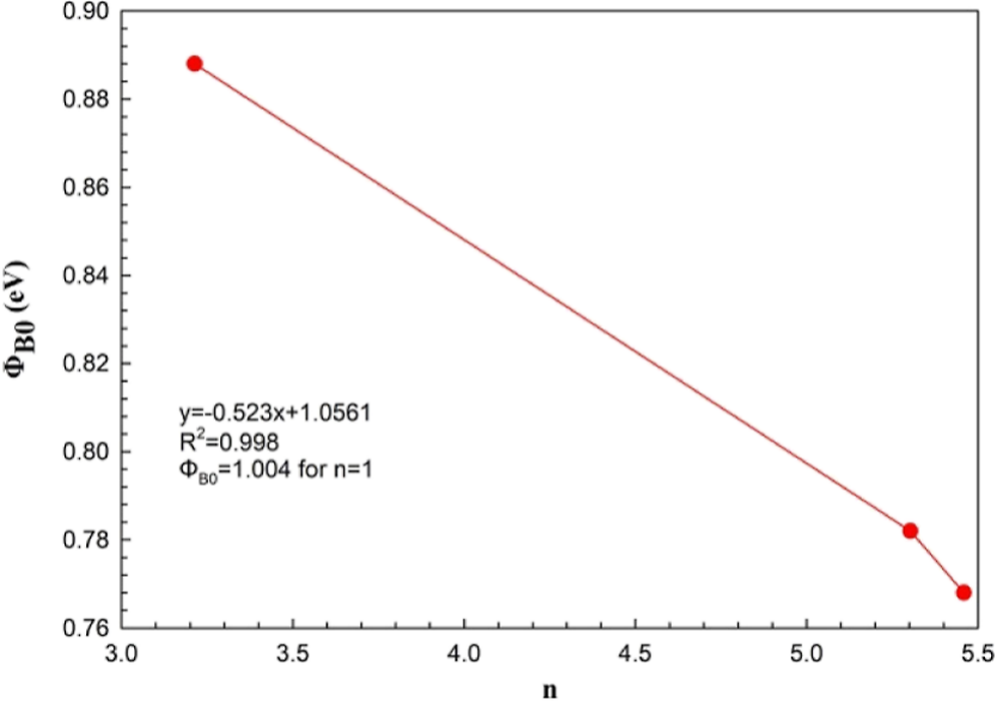
Φ_B0_ vs *n* plot of the Au/3% Gr-doped PVA/n-type Si diode.

As illustrated in Figure 5, the relationship between Φ_B0_ and *n* is defined with the equation of Φ_B0_ (*n*) = (−0.0523*n* + 1.0561), and the value of Φ_B0_ for the ideal situation (*n* = 1) is predicted as 1.004 eV. The existence of graphene is related to the following characteristics: PVA interlayer, thickness, depletion layer width (*W*_d_), donor atom doping concentration (*N*_d_), *N*_ss_, and barrier inhomogeneity.^7,8,30−33^

Electron–hole (e–h) pair generation, electron and hole recombination, hole transit, hole trapping, and hole generation on the surfaces of n-type Si semiconductors constitute some of the significant effects of irradiation exposure. The proportion of ray energy that is transformed into electron–hole pairs relies on the characteristics of Gr-doped-PVA because the length of the depletion area is a crucial factor in the generation of electron–hole pairs. In addition, the technique for producing e–h pairs demonstrates how effectively e-h pairs are produced in graphene-doped PVA-based diodes. It is crucial to understand that the fundamental idea behind semiconductor radiation detectors is the generation of e-h pairs in the depletion area.^34−38^

We used Norde and Cheung functions in addition to the TE theory to account for the impact of *R*_s_ and Φ_B0_. The following relationships may be used to compute the three fundamental diode parameters, such as Φ_B0_, *R*_s_, and *n*, in a second way at sufficiently high forward bias voltages using the Cheung functions.

**Figure 6:**
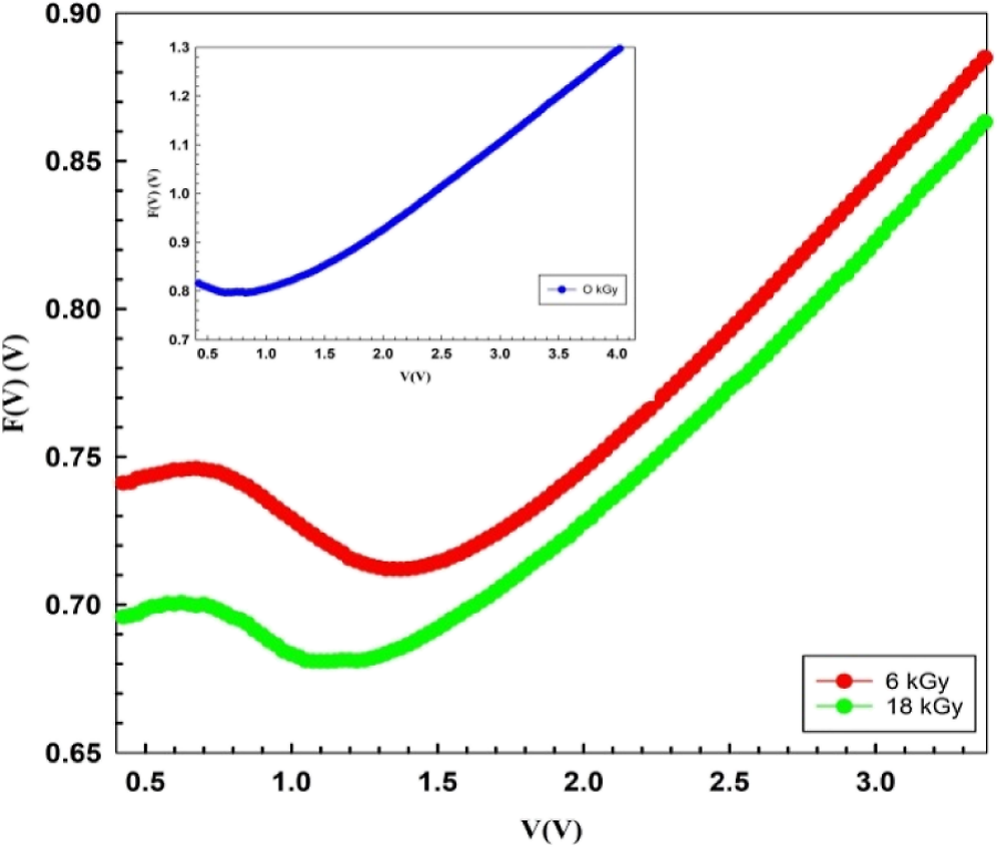
*F*(*V*) vs *V* plots of the Au/3% Gr-doped PVA/n-type Si diode.

First, the Norde function (*F*(*V*)) is a different way to determine the values of *R*_s_ and Φ_B0_, and this function is stated as follows^32,39^
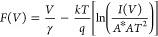
5where γ must be a positive integer greater than *n*, which has no dimensions. Equation 5 is used to generate the *F*(*V*) versus *V* plots before and after the β-irradiation, and they are displayed in Figure 6. The lowest point of *F*_o_(*V*) is shown by the concave section in Figure 6, and the voltage associated with it is *V*_o_. Finally, by utilizing the established *F*_o_(*V*) and *V*_o_ values and the related equation below, it is feasible to infer Φ_B0_ and *R*_S_ values.
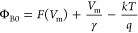
6a
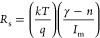
6bThe values of *R*_s_ and Φ_B0_ may be calculated via the *F*(*V*) vs *V* plots using the *I*_m_ and *V*_m_ values that correspond to the lowest value of this plot as illustrated in Figure 6.

Second, according to the Cheung functions, the following relationships may be used to compute the fundamental three SD parameters, namely, *R*_s_, *n*, and Φ_B0_, at sufficiently high forward bias voltages.
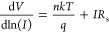
7a
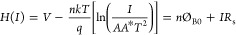
7bFigure 7a–c shows the d*V*/dln(*I*) and *H*(*I*) vs *I* plots for a diode before and after the β-irradiation using eqs 7a and 7b.

**Figure 7:**
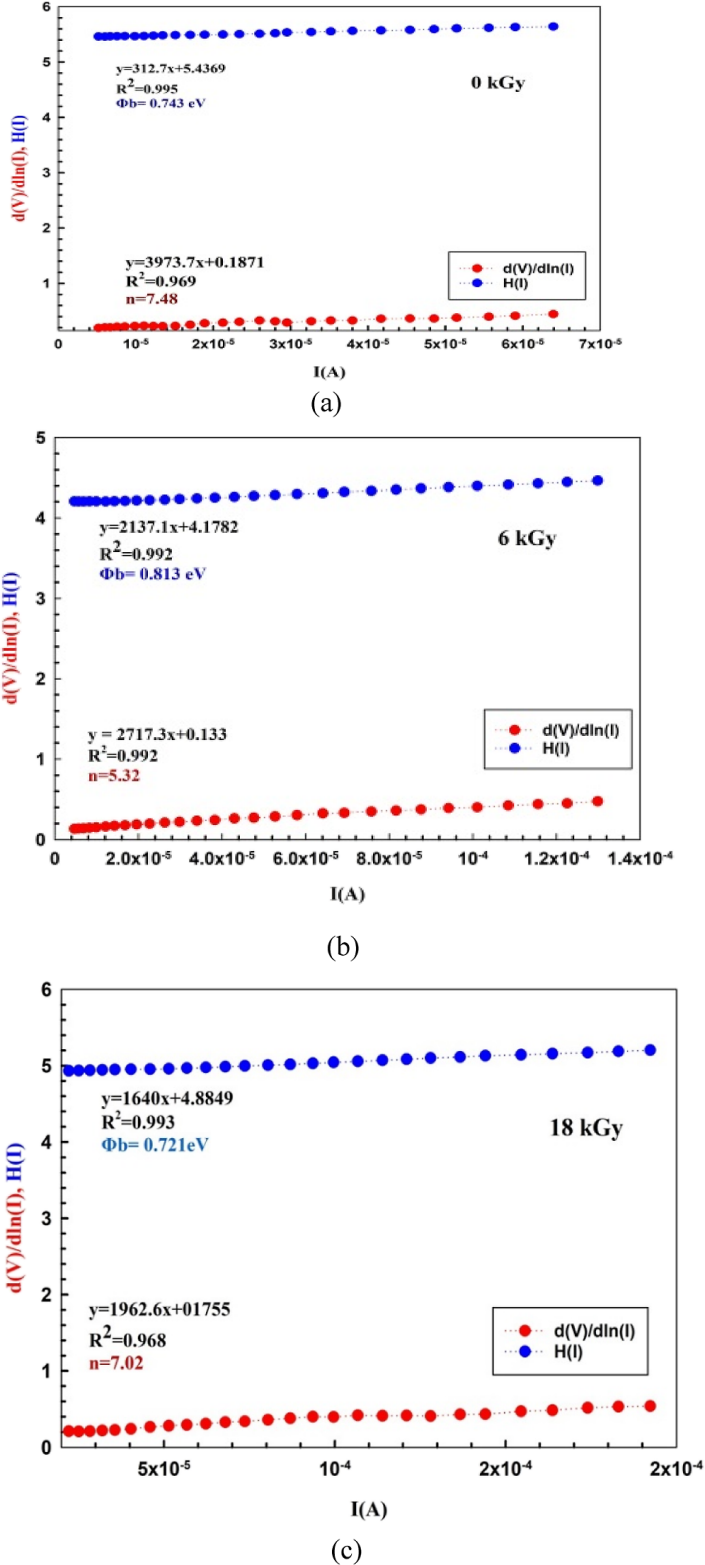
d*V*/dln(*I*) and *H*(*I*) versus *I* plots the of Au/3% Gr-doped PVA/n-type Si diode (a) for 0 kGy (b) 6 kGy (c) for 18 kGy.

The intersection and slopes of the d*V*/dln(*I*) vs *I* plots were calculated using eq 7a to obtain the *n* and *R*_s_ values. Next, *H*(*I*) versus *I* plots were drawn using the *n* values in eq 7b, and the slope and intersection point of these plots were utilized to calculate the values of *R*_s_ and Φ_B0_. To compare the results from TE, Norde, and Cheung’s techniques, the fundamental electrical parameters (*n*, *R*_s_, and Φ_B0_) of the diode were reported in Table 2. Due to the different methodologies, these methods are used to derive the voltage-dependent parameters; as indicated in Table 2, there are variations in the results.

**Table 2:** Au/3% Gr-Doped PVA/n-type Si Diode’s Electrical Parameters Using the TE, Cheung, and Norde Functions

irradiation doses (kGy)	*I*–*V*			Cheung-I		Cheung-II		Norde	
	*n*	Φ_B0_ (eV)	*R*_s_ (kΩ)	*n*	*R*_s_ (kΩ)	Φ_B0_ (eV)	*R*_s_ (kΩ)	Φ_B0_ (eV)	*R*_s_ (kΩ)
0	3.21	0.888	5.25	7.22	3.97	0.74	3.22	0.905	16.12
6	5.30	0.782	3.47	5.14	2.72	0.813	2.14	0.859	5.31
18	5.46	0.768	2.63	6.78	1.96	0.721	1.64	0.792	2.49

Calculating radiation-induced surface states in the band gap requires obtaining the voltage-dependent ideality factor (*n*(*V*)) and the effective BH (Ø_e_). When the *R*_s_ impact is ignored, the *n*(*V*) and (Ø_e_) may be derived using eqs 8a and 8b.^39,40^ Thus, by accounting for *n*(*V*) and (Ø_e_), the *N*_ss_ formula may be stated as in eq 8d.^41^ In these calculations, the (*V* – *IR*_s_) expression should be used rather than the (*V*) expression if the R_s_ effect is taken into account. The terms ε_s_ and ε_i_ in these equations stand for the permittivity of the interlayer and semiconductor, respectively. For this diode, the polymer interlayer (graphene–PVA) thickness was found as 404 nm.^42^ On the contrary, it is known that eq 8c gives the energy formula from the midgap (*E*_ss_) toward the upper edge of the conduction band (*E*_c_).

With the creation of a diode structure, increasing numbers of *N*_ss_ may form between an interlayer and a semiconductor positioned near the semiconductor’s bandgap. These generally result from flaws such as oxygen vacancies, dangling limits that rely on the chemical makeup of the interfacial layer, and other irregularities in the periodic lattice.^21,41,43,44^ The use of forward-bias *I*–*V* (current–voltage) characteristics, along with consideration for the voltage-dependence of the ideality factor (*n*) and built-in potential Ø_B0_ using a voltage-dependent Ø_e_ (effective BH) and the equation for *n* established by Card and Rhoderick, enables the determination of the energy-dependent profile of interface states (*N*_ss_)
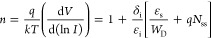
8a
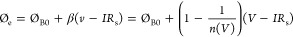
8b
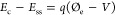
8c
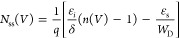
8dwhere *W*_D_ is the width of the depletion layer, and ε_s_ (=11.80 for Si) and ε_i_ (=593) are the electrical permittivity of the semiconductor and interlayer, respectively. δ is the interlayer thickness, which is computed from the interfacial layer capacitance (*C*_i_ = εε_o_*A*/δ_i_) as 404 nm. On the other hand, for n-type Si semiconductors, *E*_c_ – *E*_ss_ = *q*(Ø_e_ – *V*) may be used to describe the energy difference between the level *N*_ss_ (*E*_ss_) and the edge of the conduction band (*E*_c_).^44^

**Figure 8:**
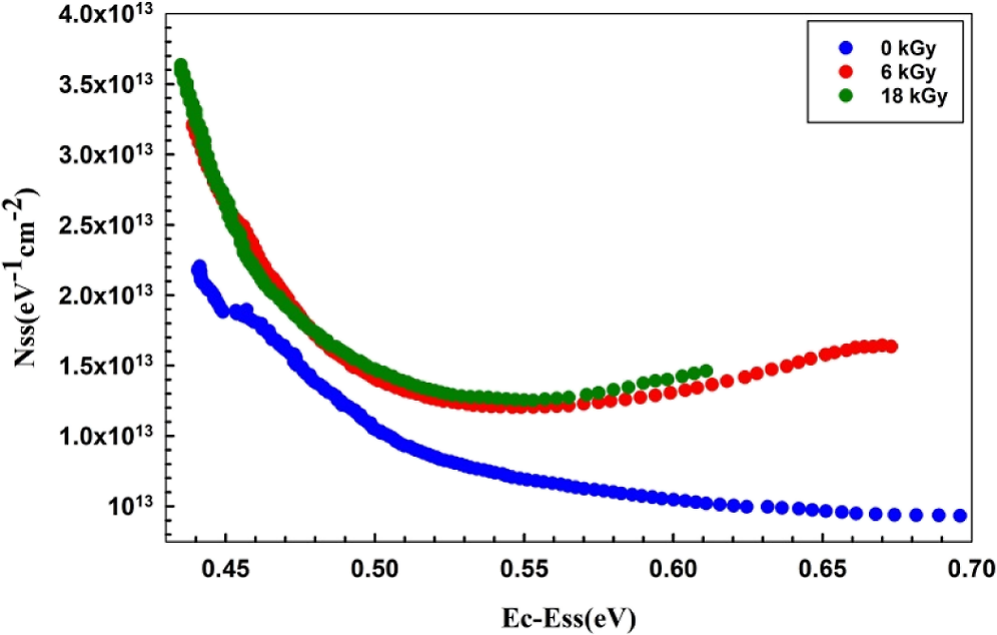
*N*_ss_ versus (*E*_c_ – *E*_ss_) plots of the Au/3% Gr-doped PVA/n-type Si diode both before and after β-irradiation.

Figure 8 shows the energy-dependent *N*_ss_ profiles before and following various beta radiation dosages. This figure shows that surface states’ density declines practically exponentially from the *E*_ss_ toward the bottom edge of the *E*_c_ both before and after irradiation. Additionally, with the increase in the radiation doses, the *N*_ss_ levels drop, as expected. The variation in *N*_ss_ behavior can be elucidated by the recombination of electron–hole (e–h) pairs influenced by radiation or the reorganization or restructuring of surface states within the band gap in the presence of an electric field. On the other hand, it can be shown that the *N*_ss_ values are rather modest when the *R*_s_ impact is taken into consideration. This outcome simply illustrates the significance of *R*_s_ in the computations.^45^

**Figure 9:**
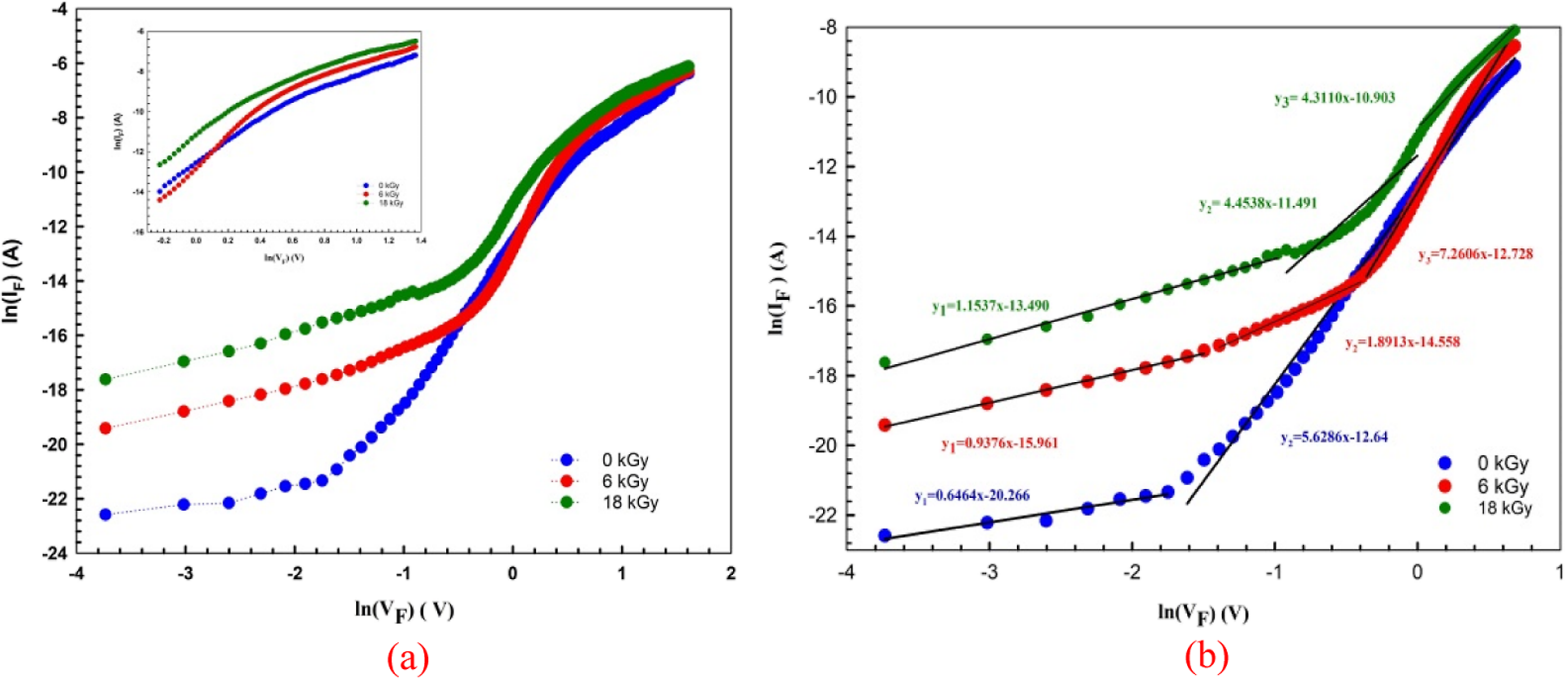
(a) ln(*I*_F_) – *V*_F_ and (b) ln(*I*_F_) – *V*_F_ linear regions of the plot for the Au/3% Gr-doped PVA/n-type Si diode before and after β-irradiation.

Additionally, the impact of the β-irradiation on the free carrier currents and current conduction mechanisms (CCMs) of the diode has been investigated at forward and reverse biases. Figure 9a,b shows the diode’s ln(*I*_F_) – *V*_F_ plots, which include several areas corresponding to various CCMs. Generally, shallow and deep traps at the metal–semiconductor contact cause an increase in the charge transfer. As can be seen in Figure 9a,b, the Au/3% Gr-doped- PVA/n-type Si diode’s (ln(*I*_F_) – *V*_F_) plot has three separate linear regions with various slopes after irradiation, as opposed to these diodes’ (ln(*I*_F_) – *V*_F_) plots, which have two distinct linear regions with different slopes before β irradiation. In the first region, these slopes are 0.6464 for 0 kGy, 0.9376 for 6 kGy, and 1.1537 for 18 kGy. In the second region, they are 5.6286 for 0 kGy, 1.8913 for 6 kGy, and 4.4538 for 18 kGy. In the third region, they are 1.8913 for 6 kGy and 4.4538 for 18 kGy. As can be observed, both linear areas have slopes (*m*) greater than 2, and the current follows the *I* ∼ *e*^*V*^*m*^^ change. These findings demonstrate that space-charge restricted current, as opposed to ohmic and trap-charge limited currents, controls the dominant CCM in the reverse bias zone. When CCMs are valid, the slope value is closer to 1 and considerably higher than 2 with increasing irradiation.

The reverse bias IR versus VR characteristics are often distinct from the forward bias *I*_F_ versus *V*_F_ features. That is because the Poole-Frenkel and Schottky emissions (PFE and SE) theories can often explain the CCM in the reverse-bias zone. The *I*_R_ is described as follows when the PFE theory is prevalent^46,47^
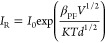
9

On the other side, the *I*_R_ is described by the following connection when the SE theory predominates.
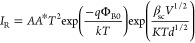
10

In eq 10, the field-lowering coefficients of β_PF_ and β_sc_ are related to each other by 2β_PF_ = β_sc_. Figure 9b shows the variations of the ln(*IR*) versus *V*_R_^1/2^ for the Au/3% Gr-doped-PVA/n-type. It can be observed that the plots of these diodes have a good linear behavior both before and after β-irradiation.

## 4 Conclusions

The effects of β-irradiation on the semiconductors’ electrical properties are very useful in a wide range of applications ranging from photodetectors to optical instruments. As explained in the present study, the structural properties of the device can be controlled using irradiation as a detector. However, ionizing radiation may pose several disadvantages such as interface traps, oxide trap charges, and defects in semiconductor devices. With the knowledge of similarities and differences between the structures and materials, we can better understand the radiation’s effects. Moreover, the radiation affects the material’s electrical parameters depending on the properties of the material. This is one of the most startling findings that we can learn by comparing the material’s data.

In this study, the impacts of the β-irradiation on the Au/3% Gr-doped PVA/n-type Si structure’s electrical parameters were investigated utilizing the *I*–*V* data, which were measured across wide ranges of voltage (±4 V) and radiation dose (0, 6, and 18 kGy). To properly assess the impacts of β-irradiation on Au/3% Gr-doped PVA/n-Si structures, the key electrical parameters such as *I*_0_, *n*, Φ_B0_, *R*_s_, RR, *N*_ss_, and *R*_sh_ were acquired utilizing the *I*–*V* characteristics of these structures before and after β-irradiation.

Additionally, the values of BH were computed as 0.888, 0.782, and 0.768 eV for 0, 6, and 18 kGy, respectively, while the values of *n* were computed as 3.21 for 0 kGy, 5.30 for 6 kGy, and 5.46 for 18 kGy. The *R*_sh_ values were computed as 0.755, 0.613, and 0.433 MΩ for 0, 6, and 18 kGy, respectively. The density of the structure increases owing to surface activation with β-irradiation compared with that of the structure that is not subjected to radiation. This was discovered by examining the structure’s energy-dependent profiles of *N*_ss_ and considering the voltage dependency of *n* and Φ_B0_. These experimental results explain that β-irradiation is more effective on the *I*–*V* characteristics. Therefore, the fabricated Au/3% Gr-doped- PVA/n-type SD can be utilized as a detector or radiation sensor.

Consequently, the following significant findings were obtained: (i) *I*_0_ values increased with the rise in the irradiation doses. Deep levels acting as generating centers and the decrease in the BH were associated with this increase. (ii) The impacts of β-irradiation led to an increase in *n* values. The widening of the depletion layer and the decline in *N*_ss_ values under β-irradiation were attributed as the causes of this behavior. The third barrier was the Si dangling bond defect caused by radiation-induced faults. (iv) As β-irradiation doses rose, *R*_s_ and *R*_sh_ levels declined.

The Au/3% Gr-doped PVA/n-type Si structure may be employed as an MPS-type detector rather than an MIS/MOS type detector because of various advantages offered by the organic/polymer interlayer in terms of being affordable, flexible, and lightweight per molecule as well as needing little energy. This finding ensures the safe operation of satellite systems, biomedical equipment, and other electronic devices running under the β-irradiation impact. To conclude, the outcomes of this study will contribute to the growing body of knowledge in the field of nanomaterials and radiation-modulated properties, paving the way for innovative technological developments.
